# Host restriction factors and p17-Driven inflammaging in HIV-1: From molecular pathogenesis to functional cure

**DOI:** 10.3934/microbiol.2026004

**Published:** 2026-03-10

**Authors:** Thomas Nitsotolis, Stelios F Assimakopoulos, Elli Kouriannidi, Maria Lagadinou, Alexia Papalexandrou, Petros Ioannou, Markos Marangos, Haralampos Milionis, Eirini Christaki

**Affiliations:** 1 Department of Internal Medicine and Infectious Diseases, Faculty of Medicine, School of Health Sciences, University of Ioannina, Ioannina, Greece; 2 Department of Internal Medicine, University of Patras Medical School, Patras, Greece; 3 3rd University Department of Internal Medicine, National and Kapodistrian University of Athens, Sotiria General Hospital for Thoracic Diseases, Athens, Greece; 4 Ionian Nephrology Center, Piraeus, Greece; 5 School of Medicine, University of Crete, Heraklion, Greece

**Keywords:** HIV-1, intrinsic immunity, p17 matrix protein, chronic inflammation, inflammaging, Serious Non-AIDS Events (SNAEs), functional cure, viral reservoir, host restriction factors, immunometabolism, bacterial translocation, gut barrier dysfunction, tight junctions, RACK1-JAK1-STAT1 pathway; glycolysis; mTOR signaling

## Abstract

Despite the widespread success of combination antiretroviral therapy (cART) in suppressing plasma viremia to undetectable levels, people living with HIV-1 (PLWH) continue to face a significantly elevated risk of chronic inflammation and Serious Non-AIDS Events (SNAEs). In this narrative review, we bridge the critical gap between molecular virology, immunometabolism, and clinical pathology by examining the complex interface of intrinsic immunity and viral persistence. We analyzed the evolutionary “arms race” between conserved host restriction factors, including TRIM5α, APOBEC3G, SAMHD1, BST-2, MX2, and SERINC, and the sophisticated viral evasion mechanisms that facilitate reservoir establishment. We further examined the role of bacterial translocation and gut barrier dysfunction in perpetuating systemic inflammation, emphasizing how HIV-1-mediated depletion of mucosal Th17 cells and disruption of tight junction proteins create a “leaky gut” that permits microbial product translocation despite suppressive therapy. Among viral proteins that may contribute to residual pathology during suppressive cART, we focused on the HIV-1 matrix protein p17, which has been proposed to function as a secreted “viral cytokine” from latent reservoirs, acting through CXCR1/CXCR2 receptors and the RACK1-JAK1-STAT1 pathway. Although primarily characterized in in vitro and ex vivo models, emerging data suggested that p17 may sustain systemic immune activation and metabolic reprogramming; however, its relative contribution compared with other viral proteins (Tat, Nef, gp120) in virologically suppressed patients remains to be fully delineated in human studies.

Furthermore, we examined how HIV-1 hijacks cellular bioenergetics by shifting host cells from oxidative phosphorylation to aerobic glycolysis. We present an integrative model that connects restriction factor biology, p17-mediated chronic inflammation, immunometabolic dysregulation, and gut barrier dysfunction into a unified pathogenic framework, distinguishing established mechanisms from working hypotheses. Last, we assessed emerging therapeutic strategies, including CRISPR/Cas9-mediated enhancement of restriction factors, modulation of the mTOR pathway, and novel “Shock and Kill” approaches, stratified by development stage and demonstrated endpoints, offering potential pathways toward a functional cure.


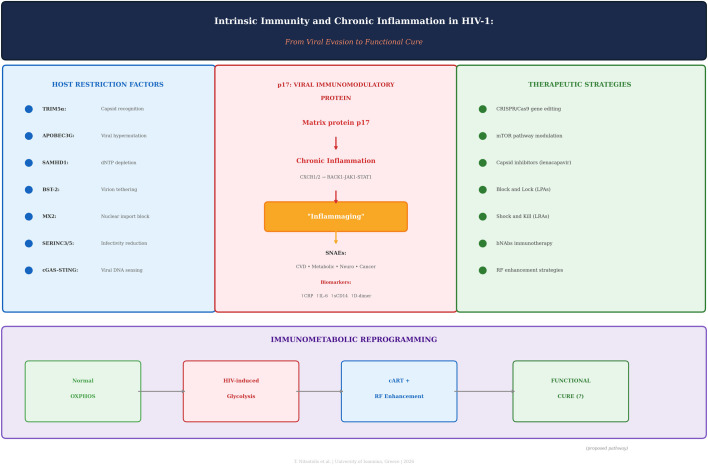


**Graphical Abstract:** *Intrinsic Immunity and Chronic Inflammation in HIV-1: From Viral Evasion to Functional Cure*.

Three interconnected thematic pillars are presented: (1) Host Restriction Factors (TRIM5α, APOBEC3G, SAMHD1, BST-2, MX2, SERINC3/5, cGAS-STING) and their antiviral mechanisms; (2) p17 as a viral immunomodulatory protein driving chronic inflammation through CXCR1/CXCR2–RACK1–JAK1–STAT1 signaling, leading to inflammaging and serious non-AIDS events (CVD, metabolic, neurological, cancer) with associated biomarkers (elevated CRP, IL-6, sCD14, D-dimer); And (3) Therapeutic Strategies encompassing CRISPR/Cas9 gene editing, mTOR pathway modulation, capsid inhibitors (lenacapavir), Block and Lock (LPAs), Shock and Kill (LRAs), bNAbs immunotherapy, and RF enhancement strategies. The bottom panel depicts immunometabolic reprogramming: The HIV-1-induced shift from normal oxidative phosphorylation (OXPHOS) to aerobic glycolysis in infected cells, and a proposed therapeutic reversal pathway through cART combined with restriction factor enhancement toward a functional cure. **Color key:** green panel (left) = host restriction factors; pink/red panel (center) = p17-driven inflammation cascade; green panel (right) = therapeutic strategies; and purple bar (bottom) = immunometabolic reprogramming. The arrows indicate the sequential pathogenic cascade from viral evasion through chronic inflammation to end-organ damage, and the proposed therapeutic reversal pathway (bottom panel) from HIV-induced glycolysis through cART with RF enhancement toward a functional cure.

## 1. Introduction

### 1.1. The clinical evolution of HIV-1 in the cART Era

Since its initial identification in the early 1980s, the Human Immunodeficiency Virus (HIV) has transitioned from an inevitably fatal infection to a manageable chronic condition [1],[2]. This paradigm shift is attributable to the development and widespread implementation of cART, which targets multiple enzymatic steps in the viral life cycle [3],[4]. Modern cART regimens can suppress plasma viral loads to undetectable levels, facilitating partial immune reconstitution, and significantly extending the life expectancy of people living with HIV (PLWH) to near-normal levels [1]–[4].

Nevertheless, the clinical efficacy of cART is constrained by inherent limitations regarding viral eradication [3]–[5]. Although treatments effectively suppress active viral replication, they cannot eliminate the latent HIV reservoir; comprising a pool of resting memory CD4+ T cells and other anatomical sanctuaries where the virus remains transcriptionally silent but can replicate upon treatment interruption [3]–[5]. This persistence necessitates lifelong adherence to therapy to avert viral rebound and maintain virological suppression [4]. Moreover, despite attaining effective virological suppression, PLWH continue to face a substantially increased risk for a range of non-HIV-related comorbidities relative to uninfected individuals, a phenomenon increasingly linked to accelerated biological aging [3]–[5]. These comorbid conditions, collectively designated Serious Non-AIDS Events (SNAEs), include cardiovascular disease, metabolic syndrome, osteopenia, renal and hepatic dysfunction, non-AIDS-defining malignancies, and neurocognitive decline [3]–[5].

### 1.2. The pathophysiological basis of SNAEs: The significance of chronic inflammation and immunometabolism

The persistence of SNAEs indicates that cART-mediated viral suppression does not eliminate viral pathogenicity [1],[4],[5]. A key factor driving this residual pathology is chronic immune activation and inflammation, sustained by the complex interaction between low-level viral protein expression, microbial translocation, and dysregulated host immune responses [3]–[5]. Specifically, viral proteins such as the HIV-1 matrix protein p17 have been reported to be released from dormant reservoirs, including lymph nodes and gut-associated lymphoid tissue (GALT), even in the absence of detectable plasma viremia [4],[5]. In vitro and limited clinical studies suggest that these proteins may act as potent pro-inflammatory mediators, potentially maintaining systemic inflammation and contributing to “inflammaging”, the accelerated biological aging of the immune system driven by chronic low-grade inflammation [3]–[5].

Furthermore, evidence underscores the vital role of immunometabolism in HIV pathogenesis [1],[5]. HIV-1 infection induces significant metabolic reprogramming in immune cells, shifting cellular metabolism from oxidative phosphorylation (OXPHOS) to aerobic glycolysis (the Warburg effect) to meet the bioenergetic demands of viral replication and chronic activation [6]. This metabolic reprogramming not only sustains viral persistence but also contributes to immune exhaustion and systemic inflammation, thereby connecting viral latency with metabolic dysregulation [5],[6].

Bacterial translocation constitutes a fundamental and well-established mechanism underlying chronic inflammation in HIV-1 infection [7],[8]. HIV-1 preferentially depletes CD4+ Th17 cells in the gut-associated lymphoid tissue (GALT), cells that are critical for maintaining intestinal barrier homeostasis through interleukin-17 (IL-17) and IL-22 secretion [7]–[9]. The depletion of these cells compromises tight junction integrity by downregulating key proteins, including claudin-1, claudin-2, occludin, and zonula occludens-1 (ZO-1), thereby increasing intestinal permeability [8],[9]. This “leaky gut” enables translocation of microbial products, particularly lipopolysaccharide (LPS), bacterial DNA (16S rDNA), and other pathogen-associated molecular patterns (PAMPs), from the intestinal lumen into systemic circulation [7],[8]. Plasma levels of microbial translocation biomarkers, including LPS-binding protein (LBP) and soluble CD14 (sCD14), and gut barrier integrity markers, including intestinal fatty acid-binding protein (I-FABP) and ZO-1, remain elevated in PLWH on suppressive cART and correlate with markers of systemic inflammation and increased cardiovascular risk [7]–[9]. Metagenomic studies have demonstrated that HIV-associated gut dysbiosis persists despite viral suppression, with the depletion of beneficial short-chain fatty acid (SCFA)-producing bacteria, such as *Faecalibacterium prausnitzii*, further exacerbating intestinal inflammation [8],[9].

The molecular architecture of enterocyte tight junctions and their disruption in HIV-1 infection have been extensively characterized [10],[11]. As comprehensively reviewed by Assimakopoulos and colleagues, the tight junction complex comprises transmembrane proteins (occludin, claudins, junctional adhesion molecules) and cytoplasmic scaffolding proteins (ZO-1, ZO-2, ZO-3) that regulate paracellular permeability [10]. HIV-1 infection disrupts this barrier at multiple levels: Direct viral effects on epithelial cells, depletion of mucosal CD4+ T cells, and cytokine-mediated disruption of tight junctions [10],[11]. Proinflammatory cytokines such as TNF-α, IFN-γ, and IL-1β activate myosin light chain kinase (MLCK), leading to actin-myosin contraction and the opening of tight junctions [10]. This multifactorial intestinal barrier dysfunction establishes a self-perpetuating cycle: Microbial translocation drives systemic immune activation, which in turn damages the gut mucosa, thereby perpetuating bacterial leakage even during effective cART [11].

Multiple HIV-1 proteins have been implicated in chronic immune activation during cART, and their relative contributions remain an area of active investigation (Table 1A). Tat is mostly recognized for its transactivation of HIV-1 transcription and its ability to induce proinflammatory cytokines through NF-κB activation; its production is largely dependent on active viral transcription and appears to be substantially reduced, though not necessarily eliminated, during effective cART [12]. Nef's immunomodulatory functions, including MHC-I downregulation and CD4 degradation, have been primarily characterized during active infection; however, studies have demonstrated that Nef can be detected in exosomes and extracellular vesicles even during suppressive therapy, suggesting a potentially underappreciated role in chronic immune dysregulation [13]. The envelope glycoprotein gp120, although capable of inducing cytokine production and endothelial dysfunction, requires specific receptor interactions (CD4, CXCR4/*CCR5*) that limit its target-cell repertoire [12],[13].

Among these viral proteins, p17 has attracted increasing attention due to several distinctive attributes: (1) It is released from latent reservoirs even in the absence of complete viral replication, as defective proviruses can produce Gag proteins (demonstrated in ex vivo and clinical detection studies) [14]; (2) it has been detected in plasma and lymphoid tissues of patients on suppressive cART in limited clinical cohorts [14],[15]; (3) it acts as a soluble “viral cytokine” by binding to CXCR1, CXCR2, and heparan sulfate proteoglycans on target cells, independent of HIV-1 entry receptors [12],[14]; and (4) in vitro studies demonstrate that it drives metabolic reprogramming and proatherogenic phenotypes in macrophages through the RACK1-JAK1-STAT1 pathway [16]. However, it should be noted that the evidence for p17 as a dominant driver of inflammaging during suppressive cART derives mostly from in vitro and ex vivo studies, with limited direct human clinical data. Prospective studies comparing the relative extracellular levels and inflammatory contributions of p17, Tat, Nef, and gp120 in virologically suppressed cohorts are needed to establish their hierarchical pathogenic significance.

### 1.3. Toward a functional cure

Attaining a definitive cure, whether a “sterilizing cure” (complete eradication of all replication-competent virus) or a “functional cure” (sustained remission without cART), necessitates a sophisticated understanding of the molecular interactions between the virus and the host [5]. Central to this interaction are the innate immune system's intrinsic antiviral restriction factors, which constitute a formidable barrier against cross-species transmission and viral propagation [5]. These proteins constitute the first line of intracellular defense, and the virus's capacity to circumvent them is a primary determinant of pathogenesis, species tropism, and the establishment of a reservoir [5]. Advances in structural biology, including cryo-electron microscopy (cryo-EM), have elucidated the precise mechanisms of capsid-restriction interactions, revealing unexpected roles for factors such as MX2 and SAMHD1 in regulating viral latency and viral core stability [5]. In this review, we critically examined these mechanisms, evaluating how modulating intrinsic immunity and correcting immunometabolic defects could be leveraged to dismantle the viral reservoir and mitigate the chronic inflammation that drives SNAEs [3],[5],[6].

## 2. Search strategy and selection criteria

This is a narrative review where we synthesize the knowledge on the interplay among intrinsic antiviral restriction factors, HIV-1 matrix protein p17-driven chronic inflammation, and emerging therapeutic strategies toward a functional cure. We searched PubMed, MEDLINE, Web of Science, and Scopus databases for peer-reviewed articles published between January 2015 and January 2026 (last search date: January 15, 2026) using the following search terms: “HIV-1,” “restriction factors,” “TRIM5α,” “APOBEC3G,” “SAMHD1,” “BST-2/tetherin,” “MX2,” “SERINC,” “p17 matrix protein,” “inflammaging,” “immunometabolism,” “Warburg effect,” “functional cure,” “latency reversal,” “CRISPR/Cas9,” and “broadly neutralizing antibodies.” We prioritized original research articles, systematic reviews, meta-analyses, and clinical trials published in English. Seminal publications preceding 2015 were included when they provided foundational evidence for key concepts. Reference lists of identified articles were manually reviewed to identify additional relevant publications. Inclusion criteria encompassed studies addressing: (a) Molecular mechanisms of host restriction factors and viral evasion, (b) extracellular effects of HIV-1 viral proteins during suppressive cART, (c) gut barrier dysfunction and microbial translocation in HIV, (d) immunometabolic reprogramming in HIV infection, and (e) preclinical or clinical studies of cure-directed interventions. Studies were excluded if they addressed HIV-2 exclusively, were conference abstracts without a peer-reviewed full-text, or were published in languages other than English. As this is a narrative review, we did not perform a formal quality assessment of the included studies; however, we prioritized higher-level evidence (randomized controlled trials, cohort studies, systematic reviews) over case reports and expert opinions. Where possible, we specified the type of evidence (in vitro, animal model, human observational, or clinical trial) supporting key claims throughout the manuscript.

## 3. HIV classification, genomic organization, and structural biology

### 3.1. Evolutionary origins and taxonomy

HIV is classified in the genus *Lentivirus* within the family *Retroviridae*, a classification that reflects the extended, often asymptomatic latency period characterizing the infection before the development of overt immunodeficiency [17]–[19]. The virus exists in two distinct types: HIV-1 and HIV-2 [17]–[19]. HIV-1, which is responsible for the global pandemic, is subdivided into four groups (M, N, O, and P), with group M being the predominant lineage, consisting of multiple subtypes (A–D, F–H, J, K) and circulating recombinant forms (CRFs) [17],[18].

Phylogenetic analyses confirm the zoonotic origins of HIV [17]–[19]. HIV-1 group M traces its lineage to Simian Immunodeficiency Virus (*SIVcpz*) found in chimpanzees (*Pan troglodytes troglodytes*), while HIV-2 is closely related to *SIVsmm* from sooty mangabeys in West Africa [17]–[19]. These evolutionary origins are critical for understanding restriction factors, as many human antiviral proteins (e.g., TRIM5α) exhibit species-specific efficacy, restricting SIV strains effectively while failing to contain HIV-1 due to viral adaptation [17]–[19]. Notably, HIV-1 group O shows greater genetic similarity to SIV strains from gorillas, indicating complex cross-species transmission events [18].

### 3.2. Genomic architecture and proteomic economy

The HIV-1 genome exemplifies remarkable genetic efficiency [20]–[22]. It comprises two identical copies of positive-sense, single-stranded RNA, each approximately 9.7 kilobases in length, flanked by Long Terminal Repeats (LTRs) [20]–[22]. The genome encodes three major structural genes (*gag*, *pol*, *env*) and an array of regulatory (*tat*, *rev*) and accessory genes (*nef*, *vif*, *vpr*, *vpu*) [20]–[22].

Complex mechanisms, including ribosomal frameshifting, regulate the expression of these genes [20]–[22]. For instance, the *pol* gene, which encodes the enzymatic machinery (Protease, Reverse Transcriptase, Integrase), is translated solely via a -1 ribosomal frameshift during translation of the *gag* transcript [20]–[22]. This mechanism ensures an approximately 20:1 stoichiometric ratio between the structural Gag proteins and the enzymatic Gag-Pol precursors, a balance vital for proper virion assembly and maturation [20]–[22].

### 3.3. The structural biology of the mature virion

The mature HIV-1 virion is a spherical particle approximately 100–120 nm in diameter, encapsulated by a host-derived lipid bilayer enriched in cholesterol and sphingolipids [23]. This envelope is adorned with trimeric glycoprotein spikes, each consisting of three surface gp120 subunits (SU) that are non-covalently associated with three transmembrane gp41 subunits (TM) [23].

#### 3.3.1. gp120 and immune evasion

The gp120 glycoprotein comprises five variable loops (V1–V5) and is extensively adorned with N-glycans [24],[25]. This “glycan shield” establishes a physical barrier that hinders neutralizing antibodies from accessing conserved viral epitopes, thereby functioning as a pivotal mechanism of humoral immune evasion [24],[25]. Cryo-EM studies have elucidated the dynamic conformational states of the Env trimer, highlighting transient open states that may be targeted by broadly neutralizing antibodies (bNAbs) [24],[25].

#### 3.3.2. gp41 and membrane fusion

The transmembrane subunit gp41 includes the fusion peptide, heptad repeats (HR1, HR2), and the membrane-proximal external region (MPER) [26]. Upon receptor engagement, gp41 experiences a significant conformational rearrangement, refolding into a six-helix bundle that facilitates the fusion of viral and host cell membranes [26].

#### 3.3.3. The capsid core

Beneath the matrix layer is the conical capsid (CA), which comprises approximately 1,500 to 2,000 p24 monomers arranged within a lattice of hexamers and pentamers [27],[28]. This core encloses the viral genome along with essential enzymes, thereby safeguarding it from cytosolic sensors and facilitating its transport to the nucleus via interactions with host factors such as cleavage and polyadenylation specificity factor 6 (CPSF6) and nucleoporins [27],[28].

### 3.4. Structural implications for restriction factor biology and p17 pathogenesis

The structural and genomic features described above have direct implications for the central themes of this review. First, the HIV-1 capsid architecture determines susceptibility to restriction factors: TRIM5α recognizes the hexameric capsid lattice, MX2 targets the capsid at nuclear pores, and capsid integrity dictates whether viral DNA is exposed to cytosolic sensors such as cGAS-STING (discussed in Section 5). Second, the Gag polyprotein processing pathway is directly relevant to p17-driven pathology: The matrix protein p17 is the N-terminal cleavage product of Gag, and its production is not contingent upon the completion of the full viral replication cycle; defective proviruses retaining intact *gag* open reading frames can produce p17 even in the absence of infectious virion assembly (discussed in Section 4). Third, the accessory genes (*vif*, *vpu*, *nef*, *vpr*) encode the viral antagonists that counteract host restriction factors, and their expression patterns during suppressive cART directly influence the balance between intrinsic immunity and viral persistence (discussed in Section 5). This structural and genomic framework thus provides the mechanistic foundation for understanding the subsequent discussion of restriction factor biology, p17-mediated chronic inflammation, and therapeutic intervention strategies.

## 4. The HIV-1 matrix protein p17: A key contributor to pathogenesis and SNAEs

Although traditionally considered a fundamental structural component of virion assembly, the HIV-1 matrix protein p17 (MA) has increasingly been recognized as an important contributor to SNAEs [29]. Its role extends beyond maintaining virion integrity; in vitro and ex vivo evidence suggests that it acts as a viral cytokine, influencing host cell signaling pathways and immunometabolism, even in the absence of active viral replication [29].

### 4.1. Structural biology and secretion

The p17 protein is a 17-kDa polypeptide derived from the N-terminus of the Gag precursor polyprotein (Pr55Gag) [29]–[31]. It undergoes myristoylation at its N-terminus, a modification that facilitates its targeting to the plasma membrane's phospholipid bilayer, where it interacts specifically with phosphatidylinositol-(4,5)-bisphosphate (PI(4,5)P2) [29]–[31]. Its major intracellular functions are to coordinate the transport of the pre-integration complex (PIC) to the nucleus and to assist in virus assembly [29]–[31]. Additionally, a significant portion of p17 is secreted into the extracellular space [29]–[31]. Notably, p17 can be detected in the plasma and tissues of patients even when viral RNA levels are suppressed by cART, implying ongoing production from latent reservoirs [29]–[31].

### 4.2. Pathogenic signaling cascades and immunometabolic reprogramming

Extracellular p17 has been shown in in vitro systems to function as a viral cytokine by binding to specific surface receptors on uninfected cells, thereby initiating adverse signaling pathways and metabolic alterations [32]:

Monocyte and macrophage activation: p17 interacts with CXCR1 and CXCR2 receptors on monocytes and macrophages, in addition to binding heparan sulfate proteoglycans (HSPGs) [33]–[35]. This interaction initiates activation of the RACK1-JAK1-STAT1 signaling pathway, leading to sustained aberrant signaling [33]–[35].

Chronic immune activation: Activation of these pathways results in elevated levels of pro-inflammatory cytokines, such as IL-1β, IL-6, and TNF-α, perpetuating systemic inflammation [33]–[35].

Atherosclerosis and vascular pathology: p17 promotes the expression of adhesion molecules (ICAM-1) and pro-thrombotic factors (Tissue Factor), thereby contributing to endothelial dysfunction and a hypercoagulable state [36],[37]. It also reduces the expression of hepatoprotective receptors, such as FXR and PPARγ, thereby exacerbating metabolic dysregulation [36],[37].

Immunometabolic dysregulation: Emerging data from in vitro models suggest that p17 signaling may drive metabolic reprogramming in immune cells, enhancing aerobic glycolysis and mitochondrial ROS production, thereby creating a pro-inflammatory microenvironment even in the absence of active viral replication [38],[39]. Whether these metabolic effects are a direct consequence of p17 signaling, an epiphenomenon of broader chronic immune activation, or an independent factor stabilizing viral reservoirs remains to be elucidated in human studies.

**Figure 1.**
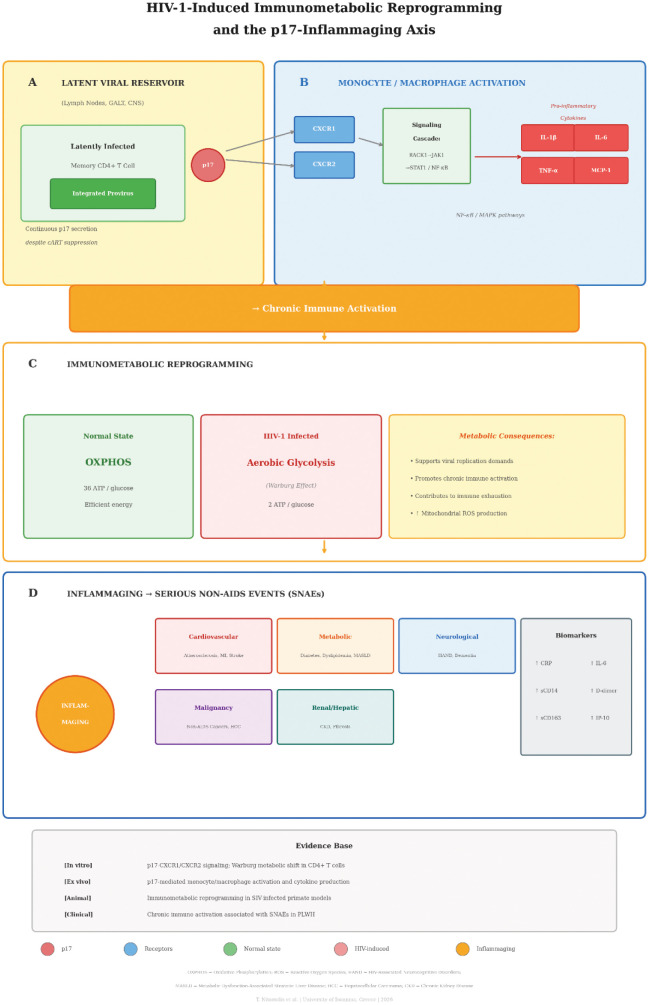
HIV-1-induced immunometabolic reprogramming and the p17-inflammaging axis. Four panels (A–D) illustrate key pathogenic mechanisms: Panel A depicts the latent viral reservoir (lymph nodes, GALT, CNS), where latently infected memory CD4+ T cells harboring integrated provirus continuously secrete the p17 matrix protein despite suppressive cART. Panel B illustrates monocyte/macrophage activation: p17 engages CXCR1/CXCR2 receptors, triggering the RACK1–JAK1–STAT1 signaling cascade and NF-κB/MAPK pathways, resulting in the production of pro-inflammatory cytokines (IL-1β, IL-6, TNF-α, MCP-1) and chronic immune activation. Panel C depicts immunometabolic reprogramming, showing the shift from normal oxidative phosphorylation (OXPHOS; 36 ATP/glucose) to aerobic glycolysis (Warburg effect; 2 ATP/glucose) in HIV-1-infected cells, with metabolic consequences including support for viral replication demands, promotion of chronic immune activation, immune exhaustion, and increased mitochondrial ROS production. Panel D illustrates how the resulting chronic low-grade inflammation (inflammaging) contributes to serious non-AIDS events (SNAEs), including cardiovascular disease, metabolic syndrome, neurodegeneration, malignancy, and renal/hepatic complications. Biomarkers of inflammaging include elevated CRP, IL-6, sCD14, D-dimer, sCD163, and IP-10. Evidence labels: p17–CXCR1/CXCR2 interaction and downstream signaling [in vitro, ex vivo]; immunometabolic shift to Warburg-type glycolysis [in vitro, animal]; and association between chronic immune activation and SNAEs [clinical, observational]. Color key: orange/yellow = latent viral reservoir; blue = monocyte/macrophage activation; green = normal metabolism; red/pink = HIV-induced metabolic shift; orange = inflammaging process; and colored rectangles = individual SNAE categories. Abbreviations: OXPHOS = Oxidative Phosphorylation; ROS = Reactive Oxygen Species; HAND = HIV-Associated Neurocognitive Disorders; MASLD = Metabolic Dysfunction-Associated Steatotic Liver Disease; HCC = Hepatocellular Carcinoma; CKD = Chronic Kidney Disease; MI = Myocardial Infarction; MCP-1 = Monocyte Chemoattractant Protein-1; IP-10 = Interferon-γ-Induced Protein 10; sCD14 = soluble CD14; sCD163 = soluble CD163.

Table 1A provides a comparative overview of extracellular HIV-1 viral proteins and their inflammatory contributions, stratified based on the evidence. Table 1B outlines the mechanisms underlying p17 pathogenesis and the associated clinical implications. Figure 1 summarizes HIV-1-induced immunometabolic reprogramming and the p17-inflammaging axis.

**Table 1A.** Comparative evidence for extracellular HIV-1 viral proteins during suppressive cart.

Viral Protein	Detection During Suppressive cART	Primary Inflammatory Mechanism	Target Receptors/Pathways	Evidence Level for Extracellular Activity During cART	Key Limitations/ Knowledge Gaps	Key References
p17 (Matrix)	Detected in plasma and lymphoid tissues by ELISA in small clinical cohorts	RACK1-JAK1-STAT1 activation; NF-κB; pro-inflammatory cytokine induction (IL-1β, IL-6, TNF-α)	CXCR1, CXCR2, HSPGs; independent of CD4/*CCR5*	In vitro +++ Ex vivo ++ Animal models + Human clinical +	Limited cohort sizes; no prospective comparative studies; detection assay variability; absence of interventional trials targeting p17	[14],[15],[29]–[35]
Tat (Transactivator)	Low-level detection in plasma; primarily transcription-dependent	NF-κB activation; direct pro-inflammatory cytokine induction; endothelial activation	Integrins, HSPGs, CXCR4 (indirect)	In vitro +++ Ex vivo ++ Animal models ++ Human clinical ±	Production is largely dependent on active transcription; levels are expected to be markedly reduced with effective cART; limited data on extracellular levels during suppression	[12]
Nef (Negative Regulatory Factor)	Detected in exosomes/ extracellular vesicles during cART	MHC-I downregulation; CD4 degradation; modulation of T-cell activation; exosome-mediated bystander effects	AP-2 complex; SERINC3/5; MHC-I	In vitro +++ Ex vivo ++ Animal models + Human clinical +	Exosomal Nef detection during cART is emerging; the functional significance of extracellular Nef during suppression is incompletely characterized	[13]
gp120 (Envelope)	Limited detection during effective cART; primarily relevant during active viremia	Cytokine induction; endothelial dysfunction; neuronal toxicity; complement activation	CD4, CXCR4, *CCR5*; requires specific receptor interactions	In vitro +++ Ex vivo ++ Animal models ++ Human clinical ±	Requires CD4/coreceptor interactions limiting target cell range; extracellular levels during effective cART are poorly characterized; complex glycosylation affects detection	[12],[13]

Evidence grading: +++ = robust evidence; ++ = moderate evidence; + = limited evidence; and ± = equivocal/insufficient evidence. This grading reflects the quantity and quality of published data, not the biological significance of the finding.

**Table 1B.** Pathogenic Effects of HIV-1 Matrix Protein p17 in Chronic HIV Infection.

Pathogenic Domain	Key Mechanisms and Clinical Implications	Evidence Source	Key References
Immune Activation	Activation of monocytes/macrophages via CXCR1/CXCR2 receptors; induction of pro-inflammatory cytokines (IL-1β, IL-6, TNF-α); signaling via NF-κB, MAPK cascades (ERK1/2, JNK, p38), and PI3K/Akt; binding to PRDX2 triggers oxidative stress responses; continuous production from viral reservoirs despite cART	In vitro, ex vivo; limited clinical detection data	[14],[15],[29],[32]–[35]
Endothelial Dysfunction	Increased expression of adhesion molecules (ICAM-1, VCAM-1, E-selectin); pro-atherogenic effects: increased ROS, reduced eNOS activity; prothrombotic state: elevated tissue factor, decreased thrombomodulin; angiogenesis via VEGF and MMP-2/MMP-9 stimulation	In vitro, animal models	[33],[36],[37]
Chronic Inflammation	Elevated inflammatory biomarkers (CRP, IL-6, sCD14, D-dimers); chronic immune activation: accelerated biological aging, telomere shortening; synergy with bacterial LPS amplifies the inflammatory response; persistent CD4+ T cell exhaustion and dysfunction	In vitro, ex vivo; human observational (biomarker correlations)	[3]–[5],[14],[15],[33]–[35]
Cardiometabolic Complications	Atherosclerosis: macrophage activation, foam cell formation, plaque instability; increased risk of acute coronary syndromes and myocardial infarction; metabolic syndrome: insulin resistance, dyslipidemia, MASLD; hypertension: increased vascular resistance, arterial stiffness	In vitro, animal models; human epidemiological associations	[36],[37]
Neurological Complications	Blood-brain barrier disruption, microglial/astrocyte activation; HIV-associated neurocognitive disorders (HAND); direct neurotoxicity: dendritic pruning, synaptic dysfunction; vascular dementia: cerebral microangiopathy	In vitro, animal models; clinical observations	[14],[82]
Neoplastic Associations	Increased incidence of non-AIDS-defining cancers; oncogenic mechanisms: chronic inflammation, immunosuppression, oxidative DNA damage; tumor angiogenesis enhancement; promotion of metastasis via MMPs and ECM degradation	In vitro; epidemiological associations	[12],[13],[33]

Note: Evidence source annotations [in vitro], [ex vivo], [animal], and [clinical] have been added to indicate the level of supporting data for each mechanism.

### 4.3. An integrative model: Connecting restriction factors, p17, gut barrier dysfunction, and immunometabolism

In the preceding sections, we describe several distinct pathogenic mechanisms that contribute to chronic inflammation in PLWH on suppressive cART. Here, we present an integrative model that connects these pathways into a hierarchical framework, distinguishing established mechanisms from hypotheses requiring further validation (Figure 1).

Established mechanisms (supported by human clinical data): (1) Despite effective cART, the latent viral reservoir persists in resting CD4+ T cells, tissue macrophages, and anatomical sanctuaries, producing intermittent viral proteins from defective proviruses. (2) HIV-1 induces profound and incompletely reversible damage to the gut mucosal barrier through Th17 cell depletion, leading to persistent microbial translocation and systemic immune activation, as demonstrated by elevated biomarkers (LPS, sCD14, I-FABP) in multiple cohort studies. (3) Chronic immune activation drives immunosenescence, T-cell exhaustion, and the inflammatory milieu associated with SNAEs.

Supported hypotheses (strong preclinical evidence, limited clinical validation): (1) HIV-1 p17, released from defective proviruses and latent reservoirs, acts as a soluble inflammatory mediator through CXCR1/CXCR2 engagement, contributing to vascular, metabolic, and neurological pathology. (2) HIV-1 induces immunometabolic reprogramming (the Warburg effect) in infected and bystander immune cells, creating a metabolic environment favorable to viral persistence and chronic activation. (3) Intrinsic restriction factors, while primarily characterized as antiviral defense mechanisms, may paradoxically contribute to chronic inflammation through sustained IFN signaling.

Working hypotheses (requiring further investigation): (1) Enhancement of intrinsic restriction factors could reduce p17 production by limiting viral protein expression from reservoir cells; however, this proposed causal link has not been directly demonstrated. (2) The potential trade-off between boosting IFN/restriction-factor pathways to reduce viral fitness and the risk of exacerbating chronic inflammation/inflammaging requires careful evaluation. (3) Immunometabolic reprogramming may serve simultaneously as a consequence of persistent viral protein expression, a stabilizing mechanism for viral reservoirs, and an independent therapeutic target, but the relative contribution of each role remains unclear.

This hierarchical framework underscores that while significant progress has been made in understanding individual pathogenic mechanisms, the causal relationships among restriction factor biology, p17-mediated inflammation, immunometabolic dysregulation, and gut barrier dysfunction remain incompletely resolved. Future studies employing systems biology approaches and well-characterized clinical cohorts will be essential to validate this integrative model.

## 5. Intrinsic restriction factors: Evolutionary dynamics

**Figure 2.**
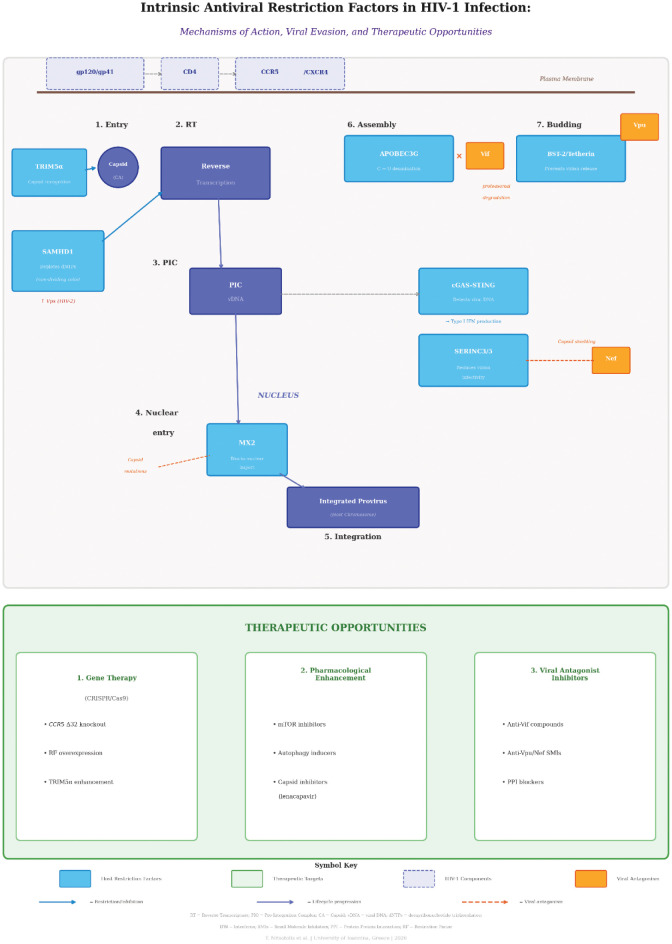
Intrinsic Antiviral Restriction Factors in HIV-1 Infection: Mechanisms of Action, Viral Evasion, and Therapeutic Opportunities. The HIV-1 replication cycle (steps 1–7: Entry, Reverse Transcription, Pre-Integration Complex formation, Nuclear Entry, Integration, Assembly, Budding) is depicted with the corresponding host intrinsic restriction factors that inhibit specific steps. Seven key restriction factors and their mechanisms are shown: TRIM5α accelerates capsid uncoating after entry; SAMHD1 depletes dNTP pools in non-dividing cells, restricting reverse transcription (antagonized by Vpx in HIV-2); APOBEC3G induces C→U deamination and hypermutation of viral DNA (counteracted by Vif-mediated proteasomal degradation); MX2 blocks nuclear entry of the pre-integration complex (evaded through capsid mutations); the cGAS-STING pathway detects cytoplasmic viral DNA and triggers type I interferon production (evaded through capsid shielding); BST-2/Tetherin prevents virion release from the plasma membrane (antagonized by Vpu); and SERINC3/5 reduces virion infectivity (counteracted by Nef). The lower panel presents three therapeutic strategy categories: (1) Gene Therapy (CRISPR/Cas9 *CCR5* knockout, restriction factor overexpression, TRIM5α/TRIMCyp enhancement); (2) Pharmacological Enhancement (mTOR inhibitors, autophagy inducers, capsid inhibitors including lenacapavir); and (3) Viral Antagonist Inhibitors (anti-Vif, Vpu, Nef compounds, small molecule inhibitors, protein–protein interaction blockers). Symbol key: blue boxes = host restriction factors; orange boxes = viral antagonism; light/peach boxes = therapeutic targets; dark purple = HIV-1 virion components; and brown = capsid/envelope structures. Solid blue arrows indicate restriction/inhibition of viral replication steps; solid dark arrows indicate lifecycle progression; and dashed red arrows indicate viral antagonism by accessory proteins. Abbreviations: RT = Reverse Transcriptase; PIC = Pre-Integration Complex; CA = Capsid; vDNA = viral DNA; dNTPs = deoxyribonucleotide triphosphates; IFN = Interferon; SMIs = Small Molecule Inhibitors; PPI = Protein–Protein Interaction; and RF = Restriction Factor.

The interaction between HIV-1 and the host immune system can be characterized as an evolutionary “arms race” [40]. The host has developed innate restriction factors, proteins constitutively expressed or induced by interferon, to inhibit viral replication [40]. In response, the virus has evolved accessory proteins to degrade or sequester these restriction factors [40]. Figure 2 provides a comprehensive schematic of these restriction factors, their mechanisms of action at specific steps of the HIV-1 replication cycle, and the corresponding viral evasion strategies.

### 5.1. TRIM5α: The capsid destabilizer

TRIM5α (Tripartite Motif-containing protein 5α) functions as a cytoplasmic restriction factor that targets the incoming viral capsid [40]–[42]. Its expression is upregulated by type I interferons (IFN-α/β), rendering it an integral component of the interferon-stimulated gene (ISG) network [40]–[42]. TRIM5α recognizes the hexagonal lattice on the surface of the HIV-1 capsid, binds to it, and triggers accelerated, aberrant capsid uncoating, exposing viral components to cytosolic sensors and inhibiting productive infection [40]–[42].

### 5.2. APOBEC3G: The lethal editor

APOBEC3G is a cytidine deaminase that operates during the reverse transcription of single-stranded viral DNA [40],[43],[44]. By catalyzing cytidine-to-uridine (C→U) deamination, it introduces guanine-to-adenine (G→A) hypermutation in the nascent proviral DNA, rendering the viral genome nonfunctional [40],[43],[44]. The HIV-1 accessory protein Vif (Viral infectivity factor) serves as the primary antagonist of APOBEC3G by recruiting the Cullin5-E3 ubiquitin ligase complex to induce its proteasomal degradation [40],[43],[44].

### 5.3. BST-2/Tetherin: The virion anchor

BST-2 (Bone Marrow Stromal Antigen 2), also known as tetherin or CD317, is an interferon-induced transmembrane protein that inhibits the release of mature virions from infected cells [40],[45],[46]. Its unique topology, possessing an N-terminal transmembrane domain and a C-terminal GPI anchor, enables it to simultaneously insert into the plasma membrane and the viral envelope, thereby physically tethering budding virions to the cell surface [40],[45],[46]. The HIV-1 accessory protein Vpu antagonizes BST-2 by displacing it from sites of viral assembly and inducing its degradation [40],[45],[46].

### 5.4. SAMHD1: The dNTP pool guardian

SAM domain and HD domain-containing protein 1 (SAMHD1) is a dNTP triphosphohydrolase (dNTPase) that restricts HIV-1 replication in myeloid cells, including macrophages and dendritic cells, as well as in quiescent CD4+ T cells [40],[47],[48]. By depleting the intracellular dNTP pool below the threshold required for efficient reverse transcription, SAMHD1 creates a nucleotide “desert” that inhibits viral DNA synthesis [40],[47],[48]. While HIV-2 encodes Vpx to degrade SAMHD1, HIV-1 lacks this antagonist, suggesting an evolutionary trade-off to maintain a “stealth” infection profile in myeloid cells [40],[47],[48].

### 5.5. MX2 and the nuclear gatekeepers

Myxovirus Resistance Protein 2, also known as MxB (MX2) is an interferon-induced GTPase that inhibits HIV-1 during the post-reverse-transcription phase, particularly at the nuclear pore complex (NPC) [40],[49],[50]. MX2 forms biomolecular condensates with nucleoporins at the NPC, trapping the HIV-1 capsid and preventing nuclear entry. This restriction depends on the capsid's interaction with host factors, such as CypA [40],[49],[50]. Capsid mutations such as N74D and A105T can confer resistance, thereby affecting viral fitness and integration site selection [40],[49],[50].

### 5.6. SERINC3 and SERINC5: Inhibitors of infectivity

The Serine Incorporator (SERINC) family of proteins, particularly SERINC3 and SERINC5, comprises transmembrane proteins that inhibit viral particle infectivity [40],[51],[52]. When incorporated into virions, SERINC3/5 inhibits viral fusion with target cells [40],[51],[52]. HIV-1 Nef antagonizes SERINC3/5 by internalizing it from the plasma membrane via the AP-2 adaptor complex, subsequently directing it toward endolysosomal degradation [40],[51],[52].

### 5.7. Innate sensing: cGAS-STING, IFI16, and ZAP

Beyond direct restrictions, the host employs sensors to detect viral components [40]. cGAS detects cytoplasmic double-stranded DNA and produces cGAMP to activate STING, thus inducing type I interferon production [40],[53]. The HIV-1 capsid shields viral DNA from detection by cGAS; however, premature uncoating, mediated by TRIM5α or pharmacological agents, such as lenacapavir, exposes the DNA, thereby activating this pathway [40],[53]. γ-Interferon-inducible protein 16 (IFI16) also functions as a DNA sensor for reverse transcription intermediates [40],[54]. Zinc-finger Antiviral Protein (ZAP) interacts with CpG-rich viral RNA; HIV-1 diminishes CpG dinucleotides to avoid detection [40],[55]. RIG-I detects viral RNA, but HIV-1 protease degrades RIG-I, aiding immune evasion [40],[56].

Table 2 provides an overview of the major intrinsic restriction factors against HIV-1. Table 3 summarizes HIV-1 accessory proteins and their corresponding counter-restriction mechanisms.

**Table 2.** Principal intrinsic restriction factors against HIV-1.

Restriction Factor	Viral Stage Target	Mechanism of Action	Viral Countermeasure (Protein or Strategy)	Primary Target Cells	Key References
TRIM5α	Post-entry/Uncoating	Recognizes capsid lattice; accelerates premature capsid disassembly; inhibits nuclear import of viral DNA	Capsid sequence polymorphisms (adaptive mutations)	All cell types	[40]–[42]
APOBEC3G	Reverse transcription	Cytidine deaminase induces G-to-A hypermutation in viral DNA; it impairs integration	Vif (proteasomal degradation via CUL5-E3 ligase)	CD4+ T cells, macrophages	[40],[43],[44]
BST-2/Tetherin	Virion release	Tethers nascent virions to the plasma membrane; prevents viral dissemination; triggers innate signaling	Vpu (displacement + degradation)	CD4+ T cells, macrophages	[40],[45],[46]
SAMHD1	Reverse transcription	dNTPase activity depletes intracellular dNTP pools; inhibits reverse transcription in non-dividing cells	Vpx (HIV-2 only; proteasomal degradation via DCAF1)	Macrophages, DCs, resting T cells	[40],[47],[48]
MX2	Nuclear import	Forms nucleoporin-containing condensates; traps viral capsids; blocks nuclear entry of PIC	Capsid mutations (N74D, A105T)	CD4+ T cells, macrophages	[40],[49],[50]
SERINC3/5	Virion infectivity	Incorporated into virions; impairs membrane fusion; reduces nascent viral particle infectivity	Nef (AP-2-mediated internalization and endolysosomal degradation)	CD4+ T cells	[40],[51],[52]
cGAS-STING	Post-uncoating/sensing	Detects cytoplasmic viral DNA; produces cGAMP to activate STING; induces type I IFN production	Capsid shielding of viral DNA during nuclear import	All cell types	[40],[53]
IFI16	Reverse transcription	Nuclear/cytoplasmic DNA sensor; detects RT intermediates; activates inflammasome and IFN responses	No well-characterized antagonist identified	CD4+ T cells, macrophages	[40],[54]
ZAP	Post-transcription	Binds CpG-rich viral RNA; recruits RNA degradation machinery	Evolutionary suppression of CpG dinucleotides in the viral genome	All cell types	[40],[55]
RIG-I	Post-transcription	Cytoplasmic RNA sensor; detects viral RNA; induces type I IFN production	HIV-1 Protease (RIG-I degradation)	pDCs, macrophages	[40],[56]

Note: The “Viral Antagonist” column header has been revised to “Viral Countermeasure (Protein or Strategy)” to distinguish antagonist proteins (e.g., Vif, Vpu, and Nef) and broader viral strategies (e.g., capsid sequence polymorphisms and CpG suppression). References have been added for each restriction factor.

**Table 3.** HIV-1 accessory proteins and counter-restriction mechanisms.

Accessory Protein	Target Host Factor(s)	Mechanism of Evasion	Additional Functions	Evidence Level	Key References
Vif	APOBEC3 family (primarily APOBEC3G)	Recruits Cullin5-E3 ubiquitin ligase complex; induces proteasomal degradation; prevents virion incorporation	Regulates cell cycle; improves viral replication efficiency	In vitro +++; Structural +++; Clinical validation ++	[40],[43]–[45]
Vpu	BST-2/Tetherin, CD4	Displaces BST-2 from assembly sites; induces CD4 degradation; manipulates ESCRT and retromer complexes	Enhances virion release; down-regulates MHC-I; inhibits NF-κB signaling	In vitro +++; Structural ++; Clinical validation +	[40],[46]
Nef	SERINC3/5, CD4, MHC-I	Removes SERINC3/5 from plasma membrane via AP-2 adaptor; prevents virion incorporation	Enhances infectivity and immune evasion; CD4 down-regulation; contributes to chronic immune dysregulation	In vitro +++; Structural ++; Clinical validation ++	[40],[51],[52]
Vpr	Multiple cellular pathways	Cell cycle arrest facilitates nuclear import; modulates DNA damage response; manipulates innate immunity	Enhances infection of non-dividing cells; modulates apoptosis	In vitro +++; Animal models ++; Clinical validation +	[40]
Vpx (HIV-2)	SAMHD1	Recruits DCAF1-containing E3 ubiquitin ligase; induces proteasomal degradation of SAMHD1	Facilitates efficient infection of myeloid cells and quiescent T cells	In vitro +++; Structural +++; Clinical validation + (HIV-2 only)	[40],[47],[48]

Evidence grading: +++ = robust evidence from multiple independent studies; ++ = moderate evidence; and + = limited/emerging evidence. “Structural” refers to high-resolution structural data (cryo-EM, X-ray crystallography) confirming the mechanism. “Chronic inflammation” in the original table was replaced with “chronic immune dysregulation” where appropriate per the reviewer's recommendation.

## 6. Therapeutic strategies targeting host-virus interactions

**Figure 3.**
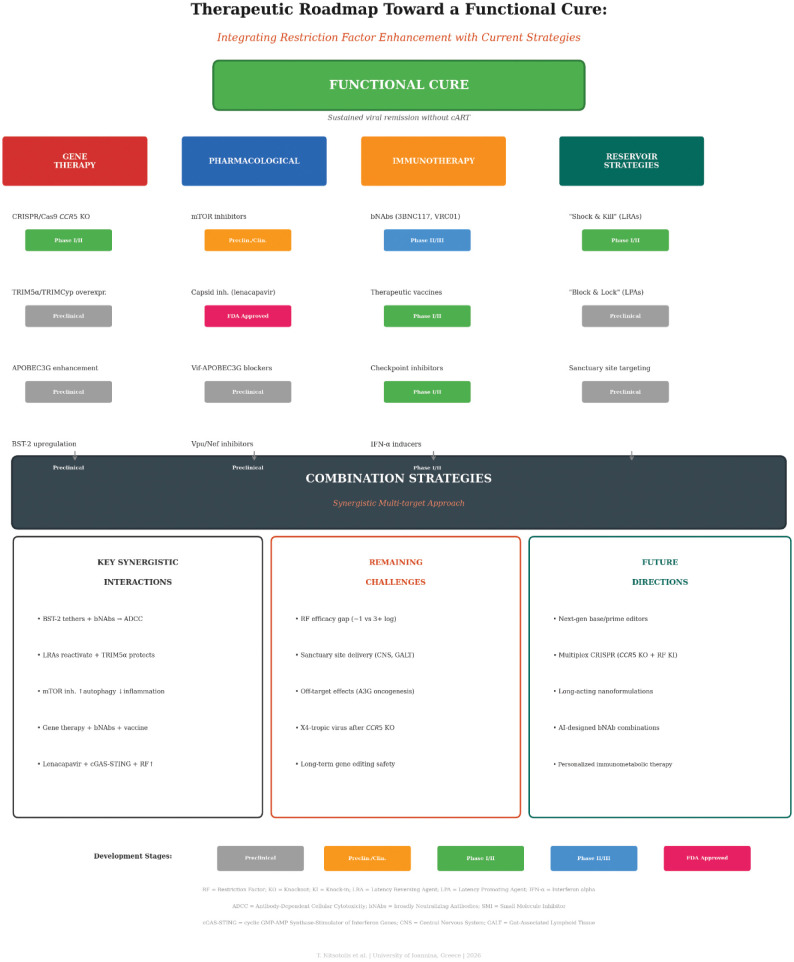
Therapeutic Roadmap Toward a Functional Cure: Integrating Restriction Factor Enhancement with Current Strategies. A multifaceted therapeutic approach to achieving a functional HIV-1 cure is presented, organized into four strategy categories that converge on a central combination approach. Gene Therapy (blue): CRISPR/Cas9 *CCR5* knockout (Phase I/II), TRIM5α/TRIMCyp overexpression (Preclinical), APOBEC3G enhancement (Preclinical), and BST-2 upregulation (Preclinical). Pharmacological (orange): mTOR inhibitors (Preclinical/Clinical observational), capsid inhibitors, including lenacapavir (FDA Approved), Vif-APOBEC3G blockers (Preclinical), and Vpu/Nef inhibitors (Preclinical). Immunotherapy (green): broadly neutralizing antibodies (Phase II/III), therapeutic vaccines (Phase I/II), checkpoint inhibitors (Phase I/II), and IFN-α inducers (Phase I/II). Reservoir Strategies (red): “Shock and Kill” using latency reversing agents (Phase I/II), “Block and Lock” using latency promoting agents (Preclinical), and sanctuary site targeting (Preclinical). All four pillars converge on a Combination Strategies platform. The lower panels summarize key synergistic interactions (BST-2 tethering + bNAbs for ADCC; LRA reactivation + TRIM5α protection; mTOR inhibition with increased autophagy and decreased inflammation; gene therapy + bNAbs + vaccine; lenacapavir + cGAS-STING + RF enhancement), remaining challenges (RF efficacy gap of ~1 vs 3+ log reduction, sanctuary site delivery to CNS and GALT, off-target effects, including A3G oncogenesis, X4-tropic virus emergence after *CCR5* knockout, and long-term gene editing safety), and future directions (next-generation base/prime editors, multiplex CRISPR combining *CCR5* knockout with RF knock-in, long-acting nanoformulations, AI-designed bNAb combinations, and personalized immunometabolic therapy). Development stage badges: grey = Preclinical; orange = Preclinical/Clinical; teal = Phase I/II; dark blue = Phase II/III; and green = FDA Approved. Abbreviations: RF = Restriction Factor; KO = Knockout; KI = Knock-in; ADCC = Antibody-Dependent Cellular Cytotoxicity; bNAbs = broadly Neutralizing Antibodies; LRA = Latency Reversing Agent; LPA = Latency Promoting Agent; CNS = Central Nervous System; GALT = Gut-Associated Lymphoid Tissue; IFN-α = Interferon alpha; A3G = APOBEC3G; SMI = Small Molecule Inhibitor.

Elucidation of the biology of restriction factors and immunometabolism has stimulated the exploration of innovative therapeutic strategies toward a functional cure [6],[40]. These strategies aim to either enhance the host's innate immune defenses or disrupt viral mechanisms that counteract them [6],[40]. Figure 3 presents the therapeutic roadmap integrating restriction factor enhancement with current cure-directed strategies.

### 6.1. Gene therapy: Re-engineering the host

The most advanced strategy involves editing the host genome to confer resistance to HIV-1, inspired by the “Berlin Patient” (Timothy Ray Brown) and the “London Patient” (Adam Castillejo) [57],[58]. Both individuals achieved long-term remission following allogeneic hematopoietic stem cell transplantation (HSCT) from donors homozygous for the *CCR5*Δ32 mutation, which renders cells impervious to R5-tropic HIV-1 [57],[58].

CRISPR/Cas9 and *CCR5* disruption: Researchers use CRISPR/Cas9 to modify the *CCR5* gene in autologous hematopoietic stem and progenitor cells (HSPCs) or CD4+ T cells [57],[58]. Phase I/II clinical trials are ongoing, with preliminary data demonstrating feasibility and safety, though long-term efficacy and complete reservoir eradication remain to be established [57],[58].

The challenge of tropism shift: A significant limitation is the presence of X4-tropic or dual-tropic viruses that utilize the CXCR4 co-receptor [59],[60]. *CCR5* ablation applies selective pressure that may facilitate the emergence of X4 variants, which are associated with swift CD4+ T cell decline and accelerated disease progression [59],[60].

Enhancing restriction factors: Researchers conducting preclinical studies are investigating the genetic overexpression of restriction factors such as TRIM5α (specifically the rhesus variant or human-rhesus chimeras like TRIMCyp) in HSPCs [61]. In preclinical models, this approach results in a “hardened” immune system, in which descendant cells remain persistently resistant to infection [61].

The clinical use of gene therapy, particularly CRISPR/Cas9, raises regulatory and ethical issues that must be resolved before widespread adoption [57], [58]. Agencies like the FDA and EMA require extensive safety data, quality controls, and phased trials with extended follow-up [57],[58]. Key concerns include: (1) Informed consent about risks; (2) fair access; (3) differences between somatic and germline editing; and (4) societal impacts of human genetic enhancement [57],[58]. WHO guidelines provide evolving frameworks for responsible research in this rapidly changing field.

### 6.2. Pharmacological modulation: Immunometabolism and mTOR inhibitors

Targeting the metabolic dependencies of HIV-1 offers a novel therapeutic avenue [6],[40]. The pharmacological induction of autophagy via mTOR inhibitors presents an alternative, non-genetic approach [62]–[65]. Rapamycin and its analogs (everolimus, temsirolimus), developed initially as immunosuppressants, target mTORC1 to augment autophagy and xenophagy [62]–[65]. In preclinical models, these inhibitors have been shown to reduce HIV-1 replication in CD4+ T cells, macrophages, and dendritic cells, and to enhance the activity of restriction factors such as TRIM5α [62]–[65]. A limited number of clinical observations in HIV-positive solid organ transplant recipients receiving everolimus have suggested possible immunological benefits, though these require confirmation in controlled trials [63]. Agents that redirect metabolism from glycolysis to oxidative phosphorylation (OXPHOS) may diminish the reservoir's size and activity, thereby counteracting the virus-induced Warburg effect [6],[62]–[65]. While mTOR inhibitors have demonstrated favorable effects on cellular senescence markers in preclinical settings, their characterization as “anti-aging” agents in the context of HIV remains premature and should be interpreted with caution pending dedicated clinical trial data.

### 6.3. Small molecule inhibitors of viral antagonists

A specific therapeutic strategy involves developing small molecules that disrupt protein-protein interactions (PPIs) between viral accessory proteins and restriction factors [66]. These include Vif-APOBEC3G interaction inhibitors that restore APOBEC3G's antiviral activity [67], Vpu-BST-2 blockers that enhance virion tethering [66], and Nef-SERINC inhibitors that restore viral restriction [66]. Additionally, capsid inhibitors like lenacapavir interact with the CA protein, accelerating premature capsid disassembly, exposing viral DNA to cGAS-STING, and augmenting restriction mechanisms [68],[69].

### 6.4. “Shock and Kill” and “Block and Lock” strategies

A central challenge to a cure is the latent viral reservoir [70],[71]. “Shock and Kill” strategies use latency-reversing agents (LRAs) to stimulate the reactivation of latent viruses (“Shock”) and facilitate immune system recognition of infected cells (“Kill”) [70],[71]. LRAs include HDAC inhibitors (vorinostat, romidepsin), PKC agonists (bryostatin-1, ingenol), BET bromodomain inhibitors, and TLR agonists [70],[71]. This approach is often conducted in conjunction with broadly neutralizing antibodies (bNAbs) [70],[71].

The “Block and Lock” alternative aims to achieve permanent silencing of the provirus through Latency Promoting Agents (LPAs), such as didehydro-Cortistatin A, or epigenetic silencers in conjunction with restriction factors, thereby maintaining the virus in a deeply latent state [72],[73]. During the “Shock” phase, enhancing restriction factors such as BST-2 to inhibit viral release or TRIM5α to prevent viral entry serves as a safeguard, ensuring that the reactivated virus cannot replenish the reservoir [70],[71].

### 6.5. Broadly neutralizing antibodies (bnabs) in combination therapy

Combining bNAb therapy with the enhancement of restriction factors presents a promising strategy [74],[75]. bNAbs target conserved HIV envelope epitopes and neutralize a wide array of viral strains [74],[75]. The major sites targeted include the CD4 binding site (antibodies: 3BNC117, VRC01), the V1/V2 loop apex (PG9, PG16), the V3-glycan supersite (10-1074, PGT121), and the membrane-proximal external region (10E8) [25],[76]. A fundamental distinction between bNAbs and cART lies in their ability to facilitate Fc-dependent functions, including Antibody-Dependent Cellular Cytotoxicity (ADCC) and Antibody-Dependent Cellular Phagocytosis (ADCP), thereby destroying HIV-infected cells [25],[74]–[76].

### 6.6. Therapeutic synergy: Integrating multiple modalities

Emerging preclinical and early-phase clinical evidence supports combining multiple therapies, such as capsid inhibitors, latency-reversing agents, and immunotherapies, to achieve synergistic effects against the viral reservoir:

Lenacapavir and cGAS-STING activation: Lenacapavir destabilizes the HIV-1 capsid, causing premature uncoating and exposing viral DNA to cytosolic sensors [68],[69]. This capsid disruption activates the cGAS-STING pathway, triggering innate immune responses, including type I interferon production [68],[69]. While lenacapavir is FDA-approved as an antiretroviral, its potential immunological benefits beyond direct antiviral activity are being investigated in ongoing studies.

LRAs for reservoir reactivation (Shock): Latency-reversing agents induce transcription from integrated proviruses, forcing latently infected cells to express viral antigens [70],[71]. When combined with capsid inhibitors, LRAs may achieve more complete reservoir exposure while preventing de novo infection of bystander cells [70],[71].

bNAbs for immune-mediated clearance (Kill): Broadly neutralizing antibodies targeting conserved HIV-1 envelope epitopes not only prevent viral entry but also mediate Fc-dependent effector functions, including ADCC and ADCP [74]–[76]. These mechanisms enable immune effector cells to eliminate virus-expressing reservoir cells. The combination of bNAbs with LRAs and capsid inhibitors creates a multi-layered attack [74]–[76].

This integrated approach represents a promising conceptual framework from single-modality interventions toward combinatorial cure strategies that simultaneously target viral latency, viral fitness, innate immunity, and adaptive immune clearance mechanisms [74]–[76].

### 6.7. Therapeutic vaccines in combination therapy

Therapeutic vaccines designed to elicit or enhance HIV-specific CD8+ cytotoxic T lymphocyte (CTL) responses could complement strategies targeting restriction factors [5],[40],[77]. Enhanced restriction factor activity would reduce viral burden and antigen load, potentially enabling vaccine-induced immune responses to gain control; analogous to “debulking” tumors before immunotherapy in oncology [5],[40],[77]. Mosaic vaccines targeting conserved viral epitopes across HIV-1 subtypes, or therapeutic vaccines based on dendritic cell priming, are in clinical development and could be rationally combined with restriction factor interventions [5],[40],[77].

Table 4 provides a comprehensive overview of therapeutic strategies targeting intrinsic restriction factors and viral antagonists, with explicit stratification by development stage and demonstrated endpoints (reservoir reduction, inflammation reduction, or post-treatment control).

**Table 4.** Therapeutic strategies targeting intrinsic restriction factors and viral antagonists.

Strategy Category	Target	Mechanism/Approach	Development Stage	Primary Demonstrated Endpoint	Clinical Potential (Projected)	Key References
Gene Therapy: CRISPR/Cas9	*CCR5* coreceptor	*CCR5*Δ32 knockout in HSPCs or CD4+ T cells; inhibits viral entry	Clinical Trials (Phase I/II)	Viral entry inhibition (in vitro); engraftment safety (clinical)	Functional cure for R5-tropic HIV-1; reservoir reduction	[57]–[60]
Gene Therapy: Overexpression	APOBEC3G/3B	CRISPR/Cas9 or synthetic activators induce gene expression	Preclinical	Hypermutation of the viral genome (in vitro)	Suppression of Vif-deficient strains; combination with cART	[43],[44],[67]
Gene Therapy: Overexpression	TRIM5α/TRIMCyp	Genetic modification of HSPCs to overexpress TRIM5α or TRIMCyp	Preclinical	Capsid restriction (in vitro/animal models)	HIV-1 resistance in engineered cells	[41],[42],[61]
Pharmacological: mTOR Inhibitors	Autophagy/Xenophagy	Rapamycin/everolimus induces autophagy; enhances HIV degradation	Preclinical; Clinical observations (transplant recipients)	Reduced HIV replication (in vitro); preliminary immunological observations (clinical)	Supplemental to cART; potential immunomodulatory benefits	[62]–[65]
Pharmacological: Small Molecules	Vif protein	Small-molecule inhibitors block Vif-APOBEC3G interaction	Preclinical/Early clinical	Restoration of APOBEC3G function (in vitro)	Combination therapy restoring innate immunity	[66],[67]
Capsid Inhibitors	HIV-1 Capsid (CA)	Lenacapavir disrupts capsid stability; exposes viral DNA to cGAS-STING	FDA Approved (antiretroviral)	Viral suppression (Phase III clinical trials)	Long-acting therapy; potential synergy with innate immunity	[68],[69]
Immunotherapy: bNAbs	Env glycoprotein	Target conserved epitopes; induce ADCC/ADCP	Clinical Trials (Phase II/III)	Viral suppression during ATI; Fc-mediated killing (clinical)	Viral control without cART; potential functional cure	[25],[74]–[76]
Block and Lock: LPAs	HIV-1 Tat-TAR axis	dCA, LEDGINs, and BRD4 modulators induce deep latency	Preclinical/Early clinical	Transcriptional silencing (in vitro/ex vivo)	Functional cure; permanent viral silencing	[72],[73]
Therapeutic Vaccines	HIV-specific CD8+ CTLs	Mosaic vaccines targeting conserved epitopes; DC-based vaccines	Clinical Trials (Phase I/II)	CTL responses (clinical); delayed rebound during ATI (some trials)	Immune-mediated post-treatment control	[5],[77]
Combination Strategies	Multiple targets	cART + capsid inhibitors + mTOR inhibitors + bNAbs + IFN induction	Conceptual/Early Preclinical	Synergistic effects (in vitro models only)	Comprehensive reservoir targeting requires clinical validation	[5],[40],[74]–[77]

Note: The “Primary Demonstrated Endpoint” column specifies the type of evidence and endpoint demonstrated to date, distinguishing in vitro, animal model, and clinical trial data. “Clinical Potential (Projected)” indicates anticipated therapeutic benefits that require further clinical validation. References are added per reviewer recommendation.

## 7. Obstacles to clinical implementation and future directions

### 7.1. The efficacy gap and implementation obstacles

While cART reduces viral load by over three log units, restriction factors typically achieve only a 0.5–1.0 log10 reduction in preclinical models [69],[78],[79]. This suppression is insufficient to control the virus alone [69],[78],[79]. The delivery of gene-editing tools or small molecules to sanctuary sites such as the central nervous system (CNS), lymph nodes, and gut-associated lymphoid tissue (GALT) remains a pharmacological challenge, particularly given that the blood-brain barrier limits entry and may leave the CNS reservoir untreated [69],[78],[79].

### 7.2. Safety and off-target effects

Gene editing involves inherent risks [80],[81]. Off-target cleavage by CRISPR/Cas9 can induce mutations in tumor suppressor genes, thereby increasing the long-term risk of cancer [80],[81]. Moreover, the persistent overexpression of restriction factors, such as APOBEC3G, may result in “off-target” deamination of the host genome, leading to somatic mutations and potential oncogenesis [80],[81]. The critical balance between antiviral efficacy and host safety necessitates thorough evaluation through long-term follow-up studies [80],[81].

### 7.3. CRISPR/Cas9-specific challenges

Beyond general safety concerns, CRISPR/Cas9-based therapies face several obstacles that warrant careful consideration:

Off-Target Mutagenesis: Despite advances in guided RNA design and high-fidelity Cas9 variants (e.g., eSpCas9 and HiFi Cas9), off-target cleavage remains a concern, particularly in ex vivo cell therapy, where edited cells undergo extensive expansion before reinfusion [80],[81]. Whole genome sequencing approaches such as GUIDE-seq, CIRCLE-seq, and DISCOVER-seq have improved off-target detection [80],[81].

Delivery Efficiency and Tissue Penetration: Delivering CRISPR components to HIV reservoirs is challenging [78],[79]. Ex vivo editing of HSPCs is feasible, but in vivo delivery to sanctuaries like the CNS, lymph nodes, and GALT is problematic [78],[79]. Viral vectors encounter immune barriers; non-viral methods exhibit variable tissue penetration [78],[79].

Mosaicism and Incomplete Editing: Achieving complete biallelic disruption (e.g., *CCR5*) in all relevant cells is complex [59],[60]. Mosaic editing, in which only some cells are modified, may provide incomplete protection and could promote viral escape [59],[60].

### 7.4. CNS reservoir access and the blood-brain barrier (BBB)

Anatomical barriers: The BBB comprises tight junction-forming endothelial cells, astrocyte end-feet, and pericytes that collectively limit the penetration of therapeutics into the CNS parenchyma [78],[79]. Most gene therapy vectors, large biologics (including bNAbs), and many small molecules exhibit poor penetration of the BBB [78],[79].

Long-Lived CNS reservoirs: HIV-1 establishes persistent infection in microglia and perivascular macrophages, which are long-lived cells that can harbor latent proviruses for decades [82]. These CNS-resident reservoir cells may contribute to viral rebound following treatment interruption, even when peripheral reservoirs have been substantially reduced [82].

Neuroinflammation and HAND: Therapeutic interventions must balance reservoir elimination with the risk of exacerbating neuroinflammation [82]. Aggressive latency reversal or immune activation in the CNS could potentially worsen HIV-associated neurocognitive disorders (HAND) or trigger immune reconstitution inflammatory syndrome (IRIS) [82].

### 7.5. Emerging solutions: Nanotechnology and advanced delivery systems

Nanotechnology delivery platforms offer promising solutions to overcome barriers in current therapies:

Lipid nanoparticles (LNPs): Building on the success of mRNA vaccines, LNPs are being developed for CRISPR/Cas9 delivery to HIV reservoirs [78],[79]. Targeting ligands may improve cell-specific delivery and reduce off-target effects [78],[79].

BBB-penetrating nanocarriers: Specialized CNS-targeted nanoparticles include transferrin receptor-targeted particles, ultrasound-opened BBB with nanocarriers, and intranasal routes bypassing the BBB via olfactory pathways [78],[79].

Cell-penetrating peptides and exosomes: Engineered cell-penetrating peptides aid CRISPR delivery, and exosome systems provide biocompatibility and tissue targeting [77].

Combination nanoformulations: Multifunctional nanoparticles capable of co-delivering LRAs, restriction factor-enhancing agents, and immune modulators may enable coordinated therapeutic interventions at reservoir sites [73],[74],[77].

The convergence of nanotechnology, gene editing, and immunotherapy is a frontier in HIV cure research, with the potential to overcome current limitations in accessing the reservoir and to improve therapeutic effectiveness [73]–[77].

## 8. Conclusions

Intrinsic antiviral restriction factors form an essential yet imperfect natural barrier against HIV-1 infection. The emergence of viral accessory proteins that neutralize these defenses highlights their crucial role in viral pathogenicity. Although cART has revolutionized HIV management, it does not eliminate the persistent viral reservoir nor mitigate the chronic inflammation caused by residual viral proteins, such as p17, in addition to notable immunometabolic dysregulation. This persistent inflammatory response significantly contributes to the serious non-AIDS events associated with the current HIV epidemic.

Strategies that leverage the potential of intrinsic immunity, through precise gene editing, pharmacological modulation of immunometabolism, or the disruption of viral antagonists, present a promising addition to current therapeutic options. By integrating “Shock and Kill” or “Block and Lock” approaches with metabolic restoration, we can aspire to re-arm the host immune system to surpass the virus's evolutionary capacity, moving closer to a functional cure and long-term health for PLWH.

### 8.1. Key findings and clinical implications

Several key findings emerge from this review with direct clinical relevance. First, intrinsic restriction factors (TRIM5α, APOBEC3G, SAMHD1, BST-2, MX2, SERINC) represent a critical but imperfect innate barrier against HIV-1, circumvented by viral accessory proteins; their therapeutic enhancement remains an active area of preclinical investigation. Second, the HIV-1 matrix protein p17, based mostly on in vitro and limited clinical detection data, appears to function as a secreted “viral cytokine” that may contribute to chronic inflammation and “inflammaging” even during suppressive cART. Third, HIV-1-induced immunometabolic reprogramming (the Warburg effect) represents a pathogenic mechanism sustaining viral persistence and a potential therapeutic target. Fourth, novel therapeutic strategies, including CRISPR/Cas9 gene editing, modulation of the mTOR pathway, capsid inhibitors, and viral antagonists, offer promising avenues to enhance intrinsic immunity, though most remain in preclinical or early clinical development. Fifth, combination approaches integrating “Shock and Kill” or “Block and Lock” paradigms with metabolic restoration and broadly neutralizing antibodies represent the most promising conceptual framework for achieving a functional cure.

For clinical practice, despite undetectable viral loads on cART, PLWH remain at elevated risk for cardiovascular disease, metabolic syndrome, and non-AIDS malignancies due to persistent inflammation driven by multiple mechanisms, including residual viral proteins and gut barrier dysfunction. Biomarkers of gut barrier dysfunction (I-FABP, sCD14, LBP) and systemic inflammation (IL-6, hsCRP, D-dimer) may warrant consideration in the assessment of virologically suppressed patients with persistent comorbidities, although their integration into routine clinical practice requires further validation. Long-acting antiretroviral agents such as lenacapavir not only improve adherence but may also confer immunological benefits through capsid destabilization and enhanced innate immune sensing; however, the clinical significance of these immunological effects remains under investigation. A multidisciplinary approach integrating infectious disease specialists, cardiologists, and metabolic specialists is essential for the optimal management of PLWH in the era of chronic HIV infection and aging.

### 8.2. Unanswered questions and future research priorities

Several critical questions remain to be addressed, which are as follows: What is the relative contribution of each viral protein (p17, Tat, Nef, gp120) to chronic inflammation during effective cART, and can their effects be therapeutically dissected? Can the enhancement of intrinsic restriction factors alone achieve clinically meaningful reductions in reservoir size, or is combination with other modalities essential? What are the long-term safety implications of CRISPR/Cas9-mediated *CCR5* disruption, particularly regarding off-target effects and immune function? How can CNS viral reservoirs be effectively targeted without exacerbating neuroinflammation or causing neurotoxicity? Will immunometabolic interventions (e.g., mTOR inhibitors) provide durable benefits, or will viral adaptation limit their long-term efficacy?

Looking forward, several priority areas warrant immediate attention. First, the development of reliable biomarkers to stratify patients by reservoir size, inflammatory burden, and likelihood of response to curative interventions remains an unmet need; validation of circulating p17 levels and restriction factor polymorphisms as predictive biomarkers for SNAEs represents a promising avenue. Second, refining delivery systems, particularly lipid nanoparticles and CNS-penetrating formulations, is essential to overcome the anatomical barriers that shield viral sanctuaries. Third, clinical trials must adopt innovative designs that enable the simultaneous evaluation of combination strategies while ensuring patient safety. Fourth, the development of therapeutic antibodies or small molecules targeting p17-CXCR1/CXCR2 interactions to mitigate chronic inflammation warrants investigation. Fifth, equitable access to emerging therapies must be prioritized to ensure that the benefits of scientific progress reach all PLWH globally, particularly those in resource-limited settings where the burden of HIV remains greatest.

In summary, the convergence of advances in molecular virology, immunometabolism, gene editing, and nanotechnology provides unprecedented opportunities to transform HIV from a lifelong condition requiring daily therapy into a curable or controllable infection. The realization of this vision will require sustained investment in basic and translational research, close collaboration between academia and industry, and unwavering commitment to the goal: A world free from HIV.

## Use of AI tools declaration

The authors used Claude (Anthropic, San Francisco, CA, USA), a large language model-based artificial intelligence assistant, solely for grammar checking, spelling correction, and language editing of the manuscript. The original intellectual content, including conceptualization, literature review, data synthesis, figure design, interpretation, and all scientific conclusions, was created exclusively by the authors. All authors reviewed and verified the final manuscript and take full responsibility for its content. The use of AI was limited to editorial assistance and did not involve content generation or scientific analysis. This disclosure is made in accordance with ICMJE recommendations for AI use in scholarly publications.
